# Polyarticular septic arthritis in an immunocompetent patient

**DOI:** 10.1308/003588413X13511609955292

**Published:** 2013-03

**Authors:** J Clements, A Dinneen, G Heilpern

**Affiliations:** Kingston Hospital NHS Trust,UK

**Keywords:** Arthritis, Infectious, Septic

## Abstract

Septic arthritis is an uncommon condition with an incidence of 2–3/100,000. It is clinically notable, however, as it is a rapidly destructive joint disease with significant associated morbidity and mortality. Polyarticular septic arthritis has an estimated incidence of 15% of all cases of infectious arthritis.

We report a case of polyarticular septic arthritis with involvement of bilateral shoulders and wrist to highlight the importance of early diagnosis and treatment as well as the high mortality rates associated with this condition. Bilateral septic shoulder arthritis poses a challenge to treat, and its significance should not be underestimated as even with early surgical intervention and aggressive antibiotic and fluid resuscitation death is a sad but perhaps not uncommon outcome. It is therefore imperative that the diagnosis of polyarticular septic arthritis is kept prominent in the physician’s mind when confronted with a patient with symptomatic polyarthralgia.

Septic arthritis is an uncommon condition with an incidence of 2–3/100,000.^1^ It is clinically notable, however, as it is a rapidly destructive joint disease with significant associated morbidity and mortality.^2^ Current literature suggests a mortality rate of 11% for monoarticular rising to 50% for polyarticular disease despite treatment.^2^ The native glenohumeral joint is involved in approximately 3% of cases.^3^

Polyarticular septic arthritis has an estimated incidence of 15% of all cases of infectious arthritis.^4^ However, it is underreported in the literature, and this can lead to misdiagnosis and delay in treatment as rhetoric suggests incorrectly that if bilateral joints are involved, it is unlikely to be septic arthritis.

We report a case of polyarticular septic arthritis with involvement of bilateral shoulders and wrist to highlight the importance of early diagnosis and treatment as well as the high mortality rates associated with this condition.

## Case history

A 94-year-old man was referred with a 3-day history of bilateral shoulder pain and left wrist pain. On examination, he was clinically well, apyrexic and was found to have had bilateral tense shoulder effusions. Cardiovascular and respiratory examinations were unremarkable. His blood results revealed a leucocytosis with a white cell count of 13.2 × 10^9^/l and a C-reactive protein level of 426mg/l. His chest x-ray showed no obvious consolidation and a urine dipstick examination was negative. Aspiration on the ward revealed frank pus from both shoulders with a positive Gram stain.

The patient was commenced on empirical antibiotics and taken for urgent arthroscopic washout. Perioperative findings revealed a large amount of frank pus in both shoulders. Minimal fluid was aspirated from his wrist and all was sent for microbiological examination. He became acutely unwell postoperatively with profound sepsis. Further investigation in the form of echocardiography revealed no cardiac valve lesions. Microbiology results were positive for *Staphylococcus aureus* in blood cultures, from both shoulder aspirates and wrist aspirate.

## Discussion

Septic arthritis of the glenohumeral joint is uncommon and there is little documentation in the literature regarding the management. Duncan and Sperling conducted a retrospective therapeutic study in 2008 that looked at the treatment of 19 patients with isolated shoulder sepsis.^5^ They concluded that prompt attention is needed to address joint infections before cartilaginous and soft-tissue damage occurs. Despite the small patient numbers, this remains one of the largest studies in on this topic. They found that where surgery was indicated there was no difference between open and arthroscopic washout, and that 78% of cases were managed satisfactorily with a single washout.^5^

A review of 23 patients in a 14-year period at one institution found that in isolated shoulder sepsis, 87% of cases had an at-risk co-morbidity^6^ whereas Lossos *et al* found the numbers to be closer to 59%.^3^ These co-morbidities are classified into arthritic disorders, systemic disorders, associated infections (including human immunodeficiency virus), malignancies and miscellaneous conditions such as drug-induced immunosuppression or intravenous drug use. There is a strong association of septic arthritis in patients with rheumatoid arthritis and this has been recognised for over 50 years.^7^ In 1993 Dubost *et al* looked at a series of 25 cases of polyarticular septic arthritis.^8^ They found that 13 of the patients had underlying rheumatoid disease and a further 9 were known to be immunocompromised. In our case, we recognise that this patient had chronic kidney disease (stage 3) but he was otherwise immunocompetent with no diagnosed underlying arthritis.

*S aureus* is well recognised as the most common causative organism in native joint infective arthritis.^6,8,9^ In this case, it was a fully sensitive strain and the difficulties encountered with resistant strands were not faced.

There is no literature guiding the management of bilateral shoulder septic arthritis. The ideal management for infected joints must include articular rest, intravenous antibiotics and consideration of drainage of the affected joint. Several options have been suggested for drainage including closed needle aspiration, arthroscopic washout or insertion of a catheter allowing for regular aspiration and irrigation of a joint.^3^

Lossos *et al* emphasised several points when the shoulder is being considered.^3^ As the shoulder is difficult to drain adequately with needle aspiration that frequent (ie twice daily), aspirations with a wide bore needle should be carried out until dry. If the patient does not improve or if there is coexistent osteomyelitis, surgical drainage is mandatory. However, they recognise that the data they collated do not allow statistically significant analysis or firm conclusions to be drawn. More recently, Cone *et al* published a review of staphylococcal infections of the shoulder and recommended early surgical intervention with appropriate antimicrobial treatment.^10^

As 15% of all cases of septic arthritis are thought to involve more than one joint,^4^ polyarticular septic arthritis has to be considered as a differential diagnosis for any patient with painful joints with associated reduced range of movement even if there are few or no systemic features of infection. This is particularly important in patients who may have confounding diagnostic hurdles. It is essential that the physician is not diverted from the diagnosis by the commonly held belief that polyarticular septic arthritis is an infrequent occurrence.

Current treatment is based on the evidence existing for one joint involvement, ie early diagnosis, parenteral broad spectrum antibiotics and consideration of surgical debridement. Given the increased mortality with polyarticular involvement, should we be managing these patients more aggressively with early and possibly repeated surgical washouts of all affected joints? Should we be considering intra-articular catheters for those patients, as in this case, who were deemed too unwell for a further anaesthetic?

## Conclusions

Bilateral septic shoulder arthritis poses a challenge to treat. Its significance should not be underestimated as even with early surgical intervention and aggressive antibiotic and fluid resuscitation death is a sad but perhaps not uncommon outcome. It is therefore imperative that the diagnosis of polyarticular septic arthritis is kept prominent in the physician’s mind when confronted with a patient with symptomatic polyarthralgia.
